# Diffusion of robot-assisted radical cystectomy: Nationwide trends, predictors, and association with continent urinary diversion

**DOI:** 10.1080/2090598X.2022.2032562

**Published:** 2022-02-16

**Authors:** Ahmed Elshabrawy, Hanzhang Wang, Furkan Dursun, Dharam Kaushik, Michael Liss, Robert S. Svatek, Ahmed M. Mansour

**Affiliations:** a Department of Urology, University of Texas (UT) Health, San Antonio, TX, USA; b UT Health San Antonio/MD Anderson Mays Cancer Center, San Antonio, TX, USA; c Urology and Nephrology Center, Mansoura University, Mansoura, Egypt

**Keywords:** Cystectomy, robotics, urinary bladder neoplasms, urinary diversion

## Abstract

**Objectives:**

To assess the utilisation trends of robot-assisted radical cystectomy (RARC), rates of performing continent urinary diversions (CUDs), and impact of diffusion of RARC on CUD rates.

**Methods:**

We investigated the National Cancer Database for patients with muscle-invasive bladder cancer (MIBC) who underwent RC between 2004 and 2015. Patients were stratified by surgical technique into open (ORC) and RARC groups, and by type of urinary diversion into continent (CUD) and ileal conduit (ICUD) groups. Linear regression models were fitted to evaluate time trends for surgery and conversion techniques. Multivariate logistic regression models were utilised to identify independent predictors of RARC and CUD.

**Results:**

A total of 14466 patients underwent RC for MIBC, of which 4914 (34%) underwent RARC. There was a significant increase in adoption of RARC from 22% in 2010 to 40% in 2015 (R^2^ = 0.96, *P* < 0.001), this was not associated with a change in the rates of CUD over the same period (*P* = 0.22). Across all years, ICUD was the primary type of urinary diversion, CUD was only offered in 12% in 2010 compared to 9.9% in 2015 (R^2^ = 0.33, *P* = 0.22). Multivariate analysis identified male gender (odds ratio [OR] 1.18, *P* = 0.03), academic centres (OR 1.74, *P* = 0.001), and lower T stage (T4 vs T2; OR 0.78, *P* = 0.03) as independent predictors of CUD, while surgical technique was not associated with odds of receiving CUD (*P* = 0.8).

**Conclusions:**

There is significant nationwide increasing trend of adoption of RARC. This diffusion was not associated with a decline in CUD, which remains significantly underutilised in both ORC and RARC groups.

**Abbreviations**

CUD: continent urinary diversion; ICD-O: International Classification of Diseases for Oncology; ICUD: ileal conduit urinary diversion; (N)MIBC: (non-)muscle-invasive bladder cancer; NAC, neoadjuvant chemotherapy; NCDB: National Cancer Database; OR: odds ratio;(O)(RA)RC: (open) (robot-assisted) radical cystectomy

## Introduction

Bladder cancer is the second most common genitourinary cancer, with an estimated incidence of nearly 81000 new cases in the United States annually [1]. Radical cystectomy (RC) with pelvic lymph node dissection remains the ‘gold standard’ treatment for muscle-invasive bladder cancer (MIBC) as endorsed by international guidelines [2]. Robot-assisted RC (RARC) has evolved as a minimally invasive alternative to open RC (ORC) with comparable oncological and survival outcomes [3–7]. Furthermore, RARC has been associated with less blood loss, faster recovery, and less hospitalisation time compared to ORC [8].

The recent rise in utilisation of RARC has raised concerns of a potential negative impact on rates of continent urinary diversions (CUDs) [9]. Urinary diversion has a great impact on health-related quality of life, as it affects urinary functions, as well as body image and emotional wellbeing [10]. Despite controversy, previous reports have associated CUD with better quality of life and overall patient satisfaction and is therefore considered the first choice in the shared decision-making process [10,11].

It has been reported that the majority of patients undergoing RC are good candidates for CUD; however, its utilisation remains limited; and the diffusion of RARC has been claimed to be contributing factor. Therefore, we aimed to assess nationwide practice patterns and time trends in RC, and its association with choice of urinary diversion techniques.

## Methods

### Data source

The National Cancer Database (NCDB) is a joint quality improvement programme between the American Cancer Society and the Commission on Cancer of the American College of Surgeons. With participation from >1500 commission on cancer accredited facilities, the NCDB captures 70% of newly diagnosed cancer cases in the United States. Data captured by the NCDB includes patient demographics and socioeconomic status, cancer staging, treatment modalities, oncological and survival outcomes. The study was exempted from review by an Institutional Review Board in accordance with the institutional regulations when working with de-identified administrative data.

### Study population

**Figure 1.**
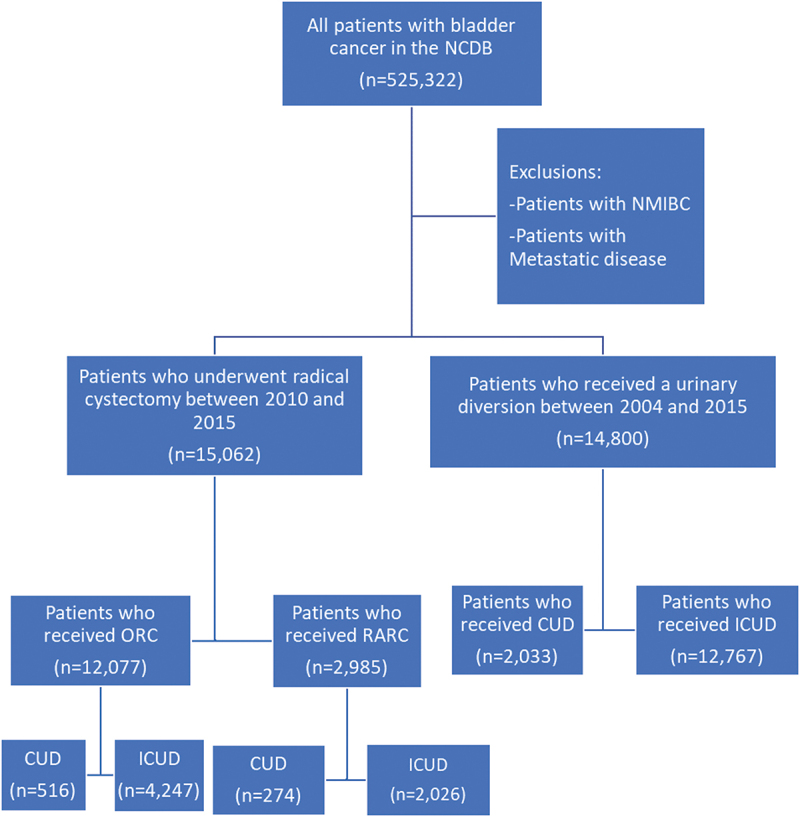
Population selection flowchart.

We queried the NCDB for patients diagnosed with bladder cancer from 2004 to 2015 (International Classification of Diseases for Oncology [ICD-O], 3rd edition topography codes C67.0–C67.9). Patients with non-metastatic cT2–T4 MIBC were identified according to the seventh edition of the American Joint Committee on Cancer (AJCC) Cancer Staging Manual. Patients were stratified according to type of urinary diversion into CUD and ileal conduit urinary diversion (ICUD) groups using codes 61 (RC plus ICUD) and codes 62 and 64 for CUD. Patients who underwent RC between 2010 and 2015 were grouped based on surgical approach into RARC and ORC, as surgical approach was not recorded by the NCDB prior to 2010. Figure 1 shows the population selection flowchart.

### Covariates and endpoints

The primary endpoint was to evaluate effect of surgical approach on utilisation of CUD in patients with MIBC treated with RC. Baseline covariates included: age, gender, year of diagnosis, race, ethnicity, type of insurance, Charlson–Deyo Comorbidity Index, insurance status, and zip-code level sociodemographic factors (annual household income, percentage of residents without high school education, and residence type according to the 2013 United States Agriculture Economic Research Service). Facility characteristics included facility type (community, academic/research, integrated cancer network, and comprehensive community cancer programmes), and facility location (grouped as Atlantic, New England, North and South East Central, North and South West Central, West Pacific and Mountain). Tumour characteristics included clinical tumour stages (T2, T3, T4), clinical nodal stage (N0, N1, ≥N2) stages, and histological variant (pure urothelial, adenocarcinoma, sarcomatoid, squamous, neuroendocrine, urothelial with micropapillary component, and others; according to the third edition of the ICD-O). Treatment modalities including surgical technique, type of urinary diversion, and receipt of neoadjuvant chemotherapy (NAC).

### Statistical analysis

Descriptive statistics were utilised to present baseline characteristics of the study groups. Pearson’s chi-square test was used to compare categorical variables between the study groups. A linear regression model was used to calculate the coefficient of determination (R^2^) and evaluate the temporal association between surgical approach and type of diversion.

Multivariate logistic regression models were fitted to identify independent predictors of receiving RARC and CUD. To construct the multivariate analyses, all covariates were tested using the univariate model, and only significant covariates were included in the multivariate model. All statistical analyses were performed using Stata® version 13 (StataCorp. [2013], College Station, TX, USA) with two-sided level of significance set at *P* < 0.05.

## Results

### Patient-related demographics

A total of 14,466 patients with cT2–T4 M0 underwent RC between 2010 and 2015. Of those, 9552 (66%) had open RC and 4914 (34%) had RARC.

Table 1 shows baseline characteristics of patients who underwent ORC and RARC. Most patients who received RARC were treated at academic centres (51.7%), resided at metro areas (79.7%), had earlier tumour and nodal stages, and approximately one-third were in the upper quartile for income (34.9%). In patients who received RARC, 2026 (88.1%) received ICUD, while 274 (11.9%) received CUD. Similarly, 4247 (89.2%) patients treated with ORC received ICUD and 516 (10.8%) received CUD.

**Table 1.** Baseline characteristics of patients who underwent ORC vs RARC.

Characteristic	ORC, *n* (%) *n* = 9552 (66)	RARC, *n* (%) *n* = 4914 (34)	Total, *n* (%) *N* = 14466 (100)	*P*
Sex				
Male	7060 (65)	3810 (35)	10870 (75.1)	
Female	2492 (69.3)	1104 (30.7)	3596 (24.9)	<0.001
Age group, years				
<60	1954 (65.5)	1029 (34.5)	2983 (20.6)	
60–69	2999 (65)	1613 (35)	4612 (31.9)	
70–79	3326 (66.7)	1658 (33.3)	4984 (34.5)	
≥80	1273 (67.4)	614 (32.6)	1887 (13)	0.15
Year of diagnosis				
2010	1816 (77.5)	527 (22.5)	2343 (16.2)	
2011	1591 (69.5)	698 (30.5)	2289 (15.8)	
2012	1518 (65.4)	803 (34.6)	2321 (16)	
2013	1527 (63.4)	881 (36.6)	2408 (16.7)	
2014	1577 (61.7)	980 (38.3)	2557 (17.7)	
2015	1523 (59.8)	1025 (40.2)	2548 (17.6)	<0.001
Race				
White	8727 (65.9)	4516 (34.1)	13243 (91.6)	
Black	562 (68.6)	257 (31.4)	819 (5.7)	
Asian	115 (68)	54 (32)	169 (1.1)	
Others	148 (62.9)	87 (37.1)	235 (1.6)	0.28
Ethnicity				
Non-Hispanic	9040 (65.8)	4681 (34.2)	13721 (97.3)	
Hispanic	262 (69.6)	114 (30.4)	376 (2.7)	0.12
Insurance				
Private	2839 (64.8)	1537 (35.2)	4376 (30.3)	
Non-Insured	251 (71.3)	101 (28.7)	352 (2.4)	
Medicaid	507 (66.7)	253 (28.3)	760 (5.3)	
Medicare	5718 (66.2)	2911 (33.8)	8629 (59.6)	
Other government	108 (65.8)	56 (34.2)	164 (1.1)	
Insurance Unknown	129 (69.7)	56 (30.3)	185 (1.3)	0.13
Charlson–Deyo Comorbidity Index				
0	6483 (65.5)	3410 (34.5)	9893 (68.4)	
1	2271 (66.8)	1126 (33.2)	3397 (23.5)	
2	603 (67.5)	290 (32.5)	893 (6.2)	
3	195 (68.9)	88 (31.1)	283 (1.9)	0.25
Annual Income, $				
<38000	1511 (69.3)	668 (30.6)	2179 (15.1)	
38000–47000	2478 (68.5)	1138 (31.5)	3616 (25.1)	
48000–62000	2708 (66.3)	1378 (33.7)	4086 (28.4)	
≥63000	2805 (62.1)	1709 (37.9)	4514 (31.4)	<0.001
NAC				
No	7545 (67.2)	3682 (32.8)	11227 (77.6)	
Yes	2007 (61.9)	1232 (38.1)	3239 (22.4)	<0.001
Facility type				
Community	393 (54.2)	332 (45.8)	725 (5)	
Comprehensive	2808 (63.5)	1616 (36.5)	4424 (30.8)	
Academic/research	5423 (68.2)	2522 (31.8)	7945 (55.3)	
Integrated network	870 (68)	409 (32)	1279 (8.9)	<0.001
Education				
≥21%	1333 (68.5)	614 (31.5)	1947 (13.5)	
13–20.9%	2534 (68.4)	1172 (31.6)	3706 (25.7)	
7–12.9%	3324 (65)	1788 (35)	5112 (35.5)	
<7%	2318 (63.6)	1324 (36.4)	3642 (25.3)	<0.001
Variant histology				
Pure urothelial	8247 (65.4)	4354 (34.6)	12601 (87)	
Adenocarcinoma	70 (73.7)	25 (26.3)	95 (0.7)	
Sarcomatoid	204 (67.8)	97 (32.2)	301 (2.1)	
Squamous	434 (73.3)	158 (26.7)	592 (4.1)	
Neuroendocrine tumour	149 (64.8)	81 (35.2)	230 (1.6)	
Urothelial with micropapillary component	157 (73.7)	56 (26.3)	213 (1.5)	
Others	291 (67)	143 (33)	434 (3)	<0.001
Location (urban/rural)				
Rural	520 (68.4)	240 (31.6)	760 (5.3)	
Urban	1734 (69.6)	756 (30.4)	2490 (17.2)	
Metro areas	7298 (65)	3918 (35)	11,216 (77.5)	<0.001
T stage				
T2	7436 (65.1)	3983 (34.9)	11,419 (78.9)	
T3	1139 (68.5)	524 (31.5)	1663 (11.5)	
T4	977 (70.6)	407 (29.4)	1384 (9.6)	<0.001
N stage				
N0	8806 (65.7)	4600 (34.3)	13406 (93.1)	
N1	335 (70)	143 (30)	478 (3.3)	
N2–3	368 (71)	150 (29)	518 (3.6)	0.007

Compared to ICUD, patients who received CUD were most frequently aged <60 years (40.3%), had less comorbidities (78.4%), were in the upper quartile of income (37.4%), treated at academic centres (64.6%), and presented at an earlier stage (82.2%) (Supplementary Table S1).

### Utilisation of RARC and CUD

**Figure 2.**
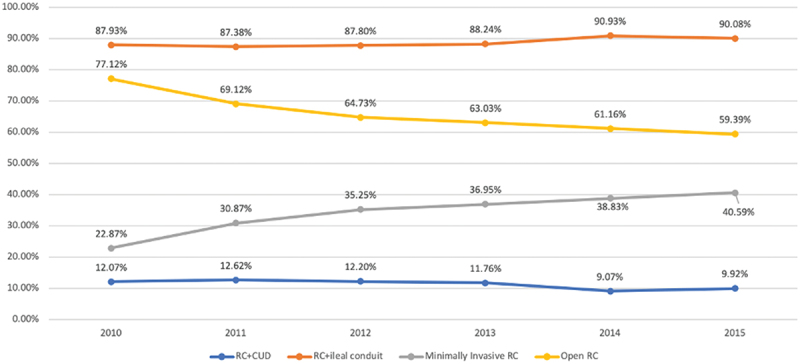
Time trends of surgical approach and urinary diversion.

There was a significant increase in adoption of RARC from 22.9% of total RCs performed in 2010 to 40.6% in 2015 (R^2^ = 0.9660, *P* < 0.001). Despite the significant increase in RARC rates, no statistically significant change was noted in rates of CUD (R^2^ = 0.33, *P* = 0.22), which remains significantly underutilised. Between 2010 and 2015, 7303 patients underwent RC with urinary diversion, of whom 828 (%11.3) had CUD and 6475 (%88.4) received ICUD (Figure 2).

### Predictors of undergoing RARC

On multivariate analysis, advancing year of diagnosis (2015 vs 2010; odds ratio [OR] 2.33, 95% CI 2.05–2.65, *P* < 0.001), male gender (OR 1.23, 95% CI 1.13–1.33, *P* < 0.001), upper quartile median income (>$63000 vs <$38000, OR 1.24, 95% CI 1.07–1.43, *P* < 0.001), receiving NAC (OR 1.24, 95% CI 1.14–1.35, *P* < 0.001) were all independent predictors for undergoing RARC. Advanced T stage (T4) was negatively associated with RARC (OR 0.82, 95% CI 0.72–0.93, *P* < 0.001) (Table 2).

**Table 2.** Multivariate logistic regression model for predictors of undergoing RARC.

Variable	Univariate analysis		Multivariate analysis	
	OR (95% CI)	*P*	OR (95% CI)	*P*
Year of diagnosis				
2010	Reference		Reference	
2011	1.51 (1.33–1.72)	<0.001	1.53 (1.34–1.75)	<0.001
2012	1.82 (1.60–2.07)	<0.001	1.84 (1.62–2.10)	<0.001
2013	1.99 (1.75–2.26)	<0.001	2.00 (1.76–2.28)	<0.001
2014	2.14 (1.89–2.43)	<0.001	2.16 (1.90–2.46)	<0.001
2015	2.32 (2.05–2.63)	<0.001	2.33 (2.05–2.65)	<0.001
Sex				
Female	Reference		Reference	
Male	1.22 (1.12–1.32)	<0.001	1.23 (1.13–1.33)	<0.001
Age group, years				
<60	Reference			
60–69	1.02 (0.93–1.13)	0.67		
70–79	0.95 (0.86–1.04)	0.26		
≥80	0.92 (0.81–1.04)	0.16		
Race				
White	Reference			
Black	0.88 (0.76–1.03)	0.11		
Asian	0.91 (0.66–1.26)	0.56		
Others	1.14 (0.87–1.48)	0.35		
Ethnicity				
Non-Hispanic	Reference			
Hispanic	0.84 (0.67–1.05)	0.13		
Charlson–Deyo Comorbidity Index				
0	Reference			
≥1	0.94 (0.87–1.01)	0.11		
Payer				
Private Insurance	Reference		Reference	
Not Insured	0.74 (0.59–0.94)	0.02	0.87 (0.68–1.11)	0.27
Medicaid	0.92 (0.78–1.09)	0.33	0.95 (0.80–1.12)	0.54
Medicare	0.94 (0.87–1.02)	0.12	0.96 (0.89–1.04)	0.37
Other Government	0.96 (0.69–1.33)	0.80	1.02 (0.73–1.42)	0.92
N/A	0.80 (0.58–1.10)	0.18	0.85 (0.61–1.17)	0.31
Income, $				
<38000	Reference		Reference	
38000–47999	1.04 (0.93–1.17)	0.50	0.99 (0.88–1.13)	0.93
48000–62999	1.15 (1.03–1.29)	0.01	1.08 (0.95–1.23)	0.27
≥63000	1.38 (1.24–1.54)	<0.001	1.24 (1.07–1.43)	<0.001
Education				
≥21%	Reference		Reference	
<7%	1.25 (1.11–1.40)	<0.001	0.96 (0.82–1.12)	0.63
13–20.9%	1.01 (0.90–1.14)	0.87	0.93 (0.82–1.05)	0.25
7–12.9%	1.17 (1.05–1.31)	<0.001	0.98 (0.86–1.12)	0.75
Facility type				
Community	Reference		Reference	
Comprehensive	0.71 (0.61–0.82)	<0.001	0.74 (0.63–0.86)	<0.001
Academic/research	0.57 (0.49–0.66)	<0.001	0.54 (0.47–0.63)	<0.001
Integrated network	0.58 (0.48–0.69)	<0.001	0.60 (0.49–0.72)	<0.001
NAC	1.26 (1.16–1.36)	<0.001	1.24 (1.14–1.35)	<0.001
Variant histology				
Pure urothelial	Reference		Reference	
Urothelial with micropapillary component	0.68 (0.50–0.92)	0.01	0.66 (0.48–0.90)	0.01
Neuroendocrine tumour	1.03 (0.79–1.36)	0.82	0.98 (0.74–1.30)	0.90
Sarcomatoid	0.9 (0.71–1.15)	0.41	0.91 (0.71–1.16)	0.43
Squamous	0.69 (0.57–0.83)	<0.001	0.71 (0.59–0.86)	<0.001
Adenocarcinoma	0.68 (0.43–1.07)	0.10	0.71 (0.44–1.12)	0.14
T Stage				
T2	Reference		Reference	
T3	0.86 (0.77–0.96)	0.01	0.90 (0.80–1.01)	0.07
T4	0.78 (0.69–0.88)	<0.001	0.82 (0.72–0.93)	0.002
N Stage				
N0	Reference		Reference	
N1	0.82 (0.67–1.00)	0.05	0.86 (0.70–1.06)	0.15
N2–N3	0.78 (0.64–0.95)	0.01	0.84 (0.68–1.02)	0.08

### Predictors of receiving CUD

Predictors of receiving CUD included male gender (OR 1.18, 95% CI 1.01–1.39, *P* = 0.03), higher income (OR 1.30, 95% CI 1.00–1.68, *P* = 0.04) and treatment in an academic/research hospital (compared to community hospitals; OR 1.74, 95% CI 1.25–2.42, *P* < 0.001). Patients were less likely to receive CUD if they had advanced disease (T4 vs T2; OR 0.78, 95% CI 0.61–0.98, *P* = 0.03). No association was found between the surgical approach and receipt of CUD (RARC vs ORC; OR 0.98, 95% CI 0.80–1.19, *P* = 0.80) (Table 3).

**Table 3.** Multivariate logistic regression model for predictors of CUD.

Variable	Univariate analysis		Multivariate analysis	
	OR (95% CI)	*P*	OR (95% CI)	*P*
Robotic vs open	1.15 (0.96–1.38)	1.15	0.98 (0.80–1.19)	0.80
Year of diagnosis				
2004	Reference		Reference	
2005	1.17 (0.85–1.61)	0.33	N/A	
2006	0.96 (0.70–1.34)	0.83	N/A	
2007	1.10 (0.81–1.49)	0.54	N/A	
2008	0.94 (0.70–1.26)	0.68	N/A	
2009	0.87 (0.65–1.17)	0.36	N/A	
2010	0.55 (0.40–0.77)	<0.001	0.45 (0.36–0.56)	<0.001
2011	0.66 (0.48–0.92)	0.01	0.69 (0.56–0.85)	<0.001
2012	0.57 (0.41–0.79)	<0.001	0.83 (0.67–1.02)	0.07
2013	0.58 (0.42–0.80)	<0.001	0.92 (0.74–1.13)	0.40
2014	0.41 (0.29–0.58)	<0.001	1.01 (0.82–1.24)	0.92
2015	0.48 (0.34–0.67)	<0.001	N/A	
Sex				
Female	Reference		Reference	
Male	2.25 (1.89–2.69)	<0.001	1.18 (1.01–1.39)	0.038
Age group, years				
<60	Reference		Reference	
60–69	0.57 (0.49–0.65)	<0.001	0.98 (0.81–1.19)	0.84
70–79	0.29 (0.25–0.34)	<0.001	0.89 (0.72–1.11)	0.29
≥80	0.17 (0.12–0.22)	<0.001	0.87 (0.67–1.12)	0.27
Ethnicity				
Non-Hispanic	Reference			
Hispanic	1.16 (0.81–1.65)	0.42		
Charlson–Deyo Comorbidity Index				
0	Reference		Reference	
≥1	0.67 (0.57–0.77)	<0.001	0.89 (0.78–1.02)	0.10
Payer				
Private insurance	Reference		Reference	
Not insured	0.70 (0.49–1.00)	0.05	0.79 (0.51–1.23)	0.29
Medicaid	0.70 (0.53–0.92)	0.01	1.01 (0.75–1.37)	0.92
Medicare	0.35 (0.31–0.40)	<0.001	0.99 (0.84–1.17)	0.88
Other Government	0.75 (0.43–1.29)	0.30	1.02 (0.58–1.81)	0.94
N/A	0.56 (0.33–0.95)	0.03	0.71 (0.38–1.35)	0.29
Income, $				
<38000	Reference		Reference	
38000–47999	1.14 (0.93–1.40)	0.22	1.11 (0.89–1.39)	0.33
48000–62999	1.22 (0.99–1.48)	0.06	1.09 (0.86–1.38)	0.45
≥63000	1.45 (1.19–1.76)	<0.001	1.30 (1.00–1.68)	0.047
Education				
≥21%	Reference		Reference	
<7%	1.27 (1.04–1.55)	0.02	0.99 (0.75–1.30)	0.92
13–20.9%	1.07 (0.87–1.31)	0.51	0.90 (0.72–1.12)	0.34
7–12.9%	1.06 (0.88–1.29)	0.53	0.94 (0.74–1.19)	0.59
Facility type				
Community	Reference		Reference	
Comprehensive	0.88 (0.68–1.13)	0.31	1.12 (0.79–1.58)	0.52
Academic/research	1.02 (0.80–1.30)	0.90	1.74 (1.25–2.42)	0.001
Integrated network	0.61 (0.43–0.85)	<0.001	1.22 (0.83–1.79)	0.31
NAC	1.04 (0.89–1.22)	0.61		
Variant histology				
Pure urothelial	Reference		Reference	
Urothelial with micropapillary component	1.14 (0.67–1.92)	0.63	0.89 (0.55–1.44)	0.63
Neuroendocrine tumour	1.43 (0.93–2.18)	0.10	1.01 (0.62–1.65)	0.96
Sarcomatoid	0.86 (0.53–1.40)	0.55	0.62 (0.37–1.03)	0.064
Squamous	0.56 (0.38–0.84)	<0.001	0.63 (0.43–0.92)	0.01
Adenocarcinoma	0.95 (0.47–1.94)	0.90	1.18 (0.57–2.46)	0.65
T Stage				
T2	Reference		Reference	
T3	0.71 (0.58–0.86)	<0.001	0.82 (0.67–1.01)	0.057
T4	0.74 (0.59–0.92)	0.01	0.78 (0.61–0.98)	0.03
N Stage				
0	Reference			
1	0.98 (0.71–1.35)	0.90		
N2–N3	0.86 (0.61–1.20)	0.38		

## Discussion

Since it was first reported in 2003 by Menon *et al*. [12], RARC has been gaining popularity in the United States. Five prospective randomised trials with a total of 541 participants aimed to compare outcomes of RARC to ORC [3–6,13]. Results from these trials showed comparable oncological outcomes with no differences in progression-free survival, recurrence-free survival, cancer-specific or overall survival between the two approaches [3,5,6]. Results from these trials have also found RARC to be associated with lower blood loss, shorter hospital length of stay, and potentially lower rates of minor complications when compared to ORC [3,8,13]. Consequently, RARC is being increasingly adopted in the urological community, with estimated rates of >50% of all performed RCs in recent multicentre series [3,14]. Previous investigations of the NCDB reported overall rates of RARC ranging from 21.4% to 34.1%, with rising rates towards later study years [15–17]. Our analysis of the NCDB found a significant near two-fold increase in adoption of RARC over 5 years, rising from 22% in 2010 to 40% in 2015.

We evaluated concurrent changes in the rates of urinary diversion with the up-trending utilisation of RARC. Between 2010 and 2015, ileal conduit was the most prevalent diversion technique, at 88% of all cases. Rates of CUD did not significantly change over the study period, with significant underutilisation of CUD with an average of 11%. Consistent with our results, previous reports from national registries identified rates of CUD between 8% and 19% [18–21]. While Lin-Brande et al. [21] identified a decline in CUD rates in the NCDB from 17.2% in 2004 to 12.1% in 2006; the report found no association between surgical approach and choice of urinary diversion.

The diffusion of the robotic approach in RC has been proposed as a contributing factor for the low utilisation of CUD. RARC is a significantly lengthier procedure than its open counterpart; and given that ICUD requires less operating time than CUD, urologists may tend to use it more often especially in elderly patients or those with comorbidities [20] Our findings did not correlate the increased adoption of RARC with any change in CUD utilisation rates, which remains significantly low.

Other possible explanations for this persistent low utilisation of CUD include low surgical volumes, lack of training opportunities, and concerns about operative time and functional outcomes [9]. These explanations are possibly supported by our analysis, showing that patients treated at academic centres were significantly more likely to receive CUD regardless of the surgical approach. Similar findings were also reported in a randomised controlled trial comparing ORC and RARC by Bochner et. al. [22], which found 55% of patients treated at academic centres received CUD regardless of surgical approach.

In addition, a recent analysis of the NCDB found that nearly 70% of RCs are occurring at low-volume centres, while only 17% of RCs were performed at a high-volume centres [23]. The correlation between high-volume centres and CUD was previously documented, as high-volume centres were associated with higher odds of receiving CUD regardless of surgical approach [21].

Techniques for robotic intracorporeal CUD have been progressing in the recent years. In addition to minimal surgical trauma, the proposed benefits of intracorporeal diversion include faster return of bowel functions, reduced fluid mobilisation, and reduced need for extensive ureteric dissection and mobilisation [24]. The International Robotic Radical Cystectomy Consortium reported outcomes of intracorporeal and extracorporeal urinary diversion in RARC and found intracorporeal urinary diversion to have lower 30- and 90-day readmission rates, lower 90-day mortality, lower rates of gastrointestinal and infectious complications in the intracorporeal group [25]. There was no significant difference in operative times or estimated blood loss between the two groups. In addition, results from several published series reported continence rates of 62–100% and 38–93% for day- and night-time continence respectively, which is similar to previously reported rates for open CUD [26,27]. While these efforts are still limited to high-volume academic institutes, and their long-term results are still underway, maturation of techniques of intracorporeal CUD could result in more adoption of CUD in the urological community.

We identified several healthcare disparities that independently impacted choice of urinary diversion regardless of surgical approach. In our cohort, female patients were significantly less likely to receive CUD. Historically, female patients were believed to be at higher risk of cancer recurrence and urinary dysfunction with an orthotopic neobladder, and thus were not offered CUD [28]. These concepts changed with maturation of techniques and improved understanding of the female pelvic musculature. However, females remain less likely to receive CUD.

In addition, patients with higher income were more likely to receive CUD regardless of type of insurance. It is suggested that patients with lower income levels are impeded from maintaining a continent diversion by lack of social and financial support [29]. They also are less likely to have received formal education, and eventually are more difficult to train for straight catheterisation if needed. On the other hand, patients with lower education levels are keener to preserve body image and would be more eager to receive CUD.

Our results showed a preference for RARC in community centres, as patients treated in academic centres were nearly half as likely to have received RARC. Possible explanations include more access to the robotic platform in the community centres, and that academic centres may possibly prioritise ORC for resident training purposes [17]. Male patients were also significantly more likely to undergo RARC than females. It has been suggested that urologists may be more familiar with performing robotic surgeries on male patients given the early widespread adoption of robot-assisted radical prostatectomies and its impact on learning curve for urological robot-assisted surgery [30].

Our results are to be interpreted within the inherent limitations of the NCDB, including retrospective data collection and possible coding errors. The NCDB does not include data about the decision-making process for CUD. Patient eligibility for CUD could not be assessed as renal function and other pertinent factors are not recorded by the NCDB. Additionally, whether the patient was offered CUD, or a provider has fully discussed diversion options or not remain unknown. Further, the NCDB records do not capture whether the decision of a planned continent diversion was altered intraoperatively nor the indication of such change if it had occurred. Data about surgeons’ volumes, experience, and number of prior RARC and/or continent diversions they had performed prior to surgery date are lacking, and such data would be valuable given the significant learning curve for both RARC and CUD. Finally, the NCDB does not differentiate between intracorporeal and extracorporeal diversion in the setting of RARC. These missing indices would help characterise providers’ role in declining rates of CUD.

Our findings suggest that the underutilisation of CUD cannot simply be explained by diffusion of the robotic approach. Rather, it is more likely to be an outcome of multiple patient-, facility-, and provider-related factors. The results also highlight the need for a standardised practice of urinary diversion and maturation of intracorporeal CUD techniques. While comparing CUD to ICUD in a randomised setting may not be feasible, optimised patient-centred functional and quality of life studies may serve as an alternative. The information gathered through these measures should assist future patients with choice of diversion. Further research is also needed to identify and address the underlying causes of underutilisation of CUD and the disparities in care that may preclude eligible patients from receiving it.

## Conclusion

RARC use has nearly doubled between 2010 and 2015, which was not associated with a decline in CUD use. On multivariate analysis, male patients and those with a higher income were more likely to receive RARC. Surgical approach was not associated with choice of urinary diversion. Patients were less likely to receive CUD if they were females, treated at community centres, or had T4 disease.

## Supplementary Material

Supplemental Material

## References

[1] Siegel RL, Miller KD, Jemal A. Cancer statistics, 2020. CA Cancer J Clin. 2020;70(1):7–30. 31912902 10.3322/caac.21590

[2] Chang SS, Bochner BH, Chou R, et al. Treatment of non-metastatic muscle-invasive bladder cancer: AUA/ASCO/ASTRO/SUO guideline. J Urol. 2017;198(3):552–559. 28456635 10.1016/j.juro.2017.04.086 PMC5626446

[3] Parekh DJ, Reis IM, Castle EP, et al. Robot-assisted radical cystectomy versus open radical cystectomy in patients with bladder cancer (RAZOR): an open-label, randomised, phase 3, non-inferiority trial. Lancet. 2018;391(10139):2525–2536. 29976469 10.1016/S0140-6736(18)30996-6

[4] Nix J, Smith A, Kurpad R, et al. Prospective randomized controlled trial of robotic versus open radical cystectomy for bladder cancer: perioperative and pathologic results. Eur Urol. 2010;57(2):196–201. 19853987 10.1016/j.eururo.2009.10.024

[5] Khan MS, Omar K, Ahmed K, et al. Long-term oncological outcomes from an early phase randomised controlled three-arm trial of open, robotic, and laparoscopic radical cystectomy (CORAL). Eur Urol. 2020;77(1):110–118. 31740072 10.1016/j.eururo.2019.10.027

[6] Bochner BH, Dalbagni G, Marzouk KH, et al. Randomized trial comparing open radical cystectomy and robot-assisted laparoscopic radical cystectomy: oncologic outcomes. Eur Urol. 2018;74(4):465–471. 29784190 10.1016/j.eururo.2018.04.030 PMC6697266

[7] Satkunasivam R, Tallman CT, Taylor JM, et al. Robot-assisted radical cystectomy versus open radical cystectomy: a meta-analysis of oncologic, perioperative, and complication-related outcomes. Eur Urol Oncol. 2019;2(4):443–447. 31277781 10.1016/j.euo.2018.10.008

[8] Novara G, Catto JW, Wilson T, et al. Systematic review and cumulative analysis of perioperative outcomes and complications after robot-assisted radical cystectomy. Eur Urol. 2015;67(3):376–401. 25560798 10.1016/j.eururo.2014.12.007

[9] Hautmann RE. Declining use of orthotopic reconstruction worldwide-what went wrong? J Urol. 2018;199(4):900–903. 29074221 10.1016/j.juro.2017.10.031

[10] Ali AS, Hayes MC, Birch B, et al. Health related quality of life (HRQoL) after cystectomy: comparison between orthotopic neobladder and ileal conduit diversion. Eur J Surg Oncol. 2015;41(3):295–299. 24913090 10.1016/j.ejso.2014.05.006

[11] World Health Organization Consensus Conference on Bladder Cancer, Hautmann RE, Abol-Enein, H, et al.; . Urinary diversion. Urology. 2007;69(1 Suppl):17–49. https://pubmed.ncbi.nlm.nih.gov/17280907/

[12] Menon M, Hemal AK, Tewari A, et al. Nerve-sparing robot-assisted radical cystoprostatectomy and urinary diversion. BJU Int. 2003;92(3):232–236. 12887473 10.1046/j.1464-410x.2003.04329.x

[13] Messer JC, Punnen S, Fitzgerald J, et al. Health-related quality of life from a prospective randomised clinical trial of robot-assisted laparoscopic vs open radical cystectomy. BJU Int. 2014;114(6):896–902. 24862633 10.1111/bju.12818

[14] Jeong W, Kumar R, Menon M. Past, present and future of urological robotic surgery. Investig Clin Urol. 2016;57(2):75–83. 10.4111/icu.2016.57.2.75 PMC4791673 26981588

[15] Ko OS, Weiner AB, Smith ND, et al. Rates and predictors of conversion to open surgery during minimally invasive radical cystectomy. J Endourol. 2018;32(6):488–494. 29620960 10.1089/end.2018.0079

[16] Hanna N, Leow JJ, Sun M, et al. Comparative effectiveness of robot-assisted vs. open radical cystectomy. Urol Oncol. 2018 Mar;36(3):88 e1–88 e9. 10.1016/j.urolonc.2017.09.018 29277584

[17] Bachman AG, Parker AA, Shaw MD, et al. Minimally invasive versus open approach for cystectomy: trends in the utilization and demographic or clinical predictors using the national cancer database. Urology. 2017;103:99–105. 28214574 10.1016/j.urology.2017.02.018

[18] Kim SP, Shah ND, Weight CJ, et al. Population-based trends in urinary diversion among patients undergoing radical cystectomy for bladder cancer. BJU Int. 2013;112(4):478–484. 23452020 10.1111/j.1464-410X.2012.11508.x

[19] Gore JL, Litwin MS. Urologic diseases in america project. Quality of care in bladder cancer: trends in urinary diversion following radical cystectomy. World J Urol. 2009;27(1):45–50. 19020881 10.1007/s00345-008-0348-y PMC2710376

[20] Bachour K, Faiena I, Salmasi A, et al. Trends in urinary diversion after radical cystectomy for urothelial carcinoma. World J Urol. 2018;36(3):409–416. 29299664 10.1007/s00345-017-2169-3

[21] Lin-Brande M, Nazemi A, Pearce SM, et al. Assessing trends in urinary diversion after radical cystectomy for bladder cancer in the United States. Urol Oncol. 2019;37(3):180 e1–180 e9. 10.1016/j.urolonc.2018.11.003 30482434

[22] Bochner BH, Dalbagni G, Sjoberg DD, et al. Comparing open radical cystectomy and robot-assisted laparoscopic radical cystectomy: a randomized clinical trial. Eur Urol. 2015;67(6):1042–1050. 25496767 10.1016/j.eururo.2014.11.043 PMC4424172

[23] Herrera JC, Ibilibor C, Wang H, et al. National trends and impact of regionalization of radical cystectomy on survival outcomes in patients with muscle invasive bladder cancer. Clin Genitourin Cancer. 2020;18(6):e762–e770. 32641262 10.1016/j.clgc.2020.05.012

[24] Chan KG, Collins JW, Wiklund NP. Robot-assisted radical cystectomy: extracorporeal vs intracorporeal urinary diversion. J Urol. 2015;193(5):1467–1469. 25686541 10.1016/j.juro.2015.02.042

[25] Ahmed K, Khan SA, Hayn MH, et al. Analysis of intracorporeal compared with extracorporeal urinary diversion after robot-assisted radical cystectomy: results from the international robotic cystectomy consortium. Eur Urol. 2014;65(2):340–347. 24183419 10.1016/j.eururo.2013.09.042

[26] Abol-Enein H, Ghoneim MA. Functional results of orthotopic ileal neobladder with serous-lined extramural ureteral reimplantation: experience with 450 patients. J Urol. 2001;165(5):1427–1432. 11342891

[27] Hautmann RE, Volkmer B, Egghart G, et al. Functional outcome and complications following ileal neobladder reconstruction in male patients without tumor recurrence. more than 35 years of experience from a single center. J Urol. 2021;205(1):174–182. 32856988 10.1097/JU.0000000000001345

[28] Lee RK, Abol-Enein H, Artibani W, et al. Urinary diversion after radical cystectomy for bladder cancer: options, patient selection, and outcomes. BJU Int. 2014;113(1):11–23. 24330062 10.1111/bju.12121

[29] Maurice MJ, Kim SP, Abouassaly R. Socioeconomic status is associated with urinary diversion utilization after radical cystectomy for bladder cancer. Int Urol Nephrol. 2017;49(1):77–82. 27696214 10.1007/s11255-016-1422-9

[30] Hayn MH, Hussain A, Mansour AM, et al. The learning curve of robot-assisted radical cystectomy: results from the international robotic cystectomy consortium. Eur Urol. 2010;58(2):197–202. 20434830 10.1016/j.eururo.2010.04.024

