# Supplementary material for: Diffusion of robot-assisted radical cystectomy: Nationwide trends, predictors, and association with continent urinary diversion
Source: Arab J Urol. 2022 Feb 16;20(3):159–67. doi: 10.1080/2090598X.2022.2032562 (PMC9354633; doi:10.1080/2090598X.2022.2032562)
Supplement: Supplemental Material [file TAJU_A_2032562_SM7892.docx]

Supplementary Table S1 Baseline characteristics of patients who received CUD vs ICUD.

| **Characteristic** | **CUD, *n* (%)**  ***n* = 2033 (13.7)** | **ICUD, *n* (%)**  ***n* = 12767 (86.3)** | **Total, *n* (%)**  ***n* = 14800 (100)** | ***P*** |
| --- | --- | --- | --- | --- |
| **Sex** |  |  |  |  |
| Male | 1815 (14.5) | 10701 (85.5) | 12516 (84.6) |  |
| Female | 218 (9.5) | 2066 (90.5) | 2284 (15.4) | <0.001 |
| **Age group, years** |  |  |  |  |
| <60 | 819 (26.8) | 2235 (73.2) | 3054 (20.6) |  |
| 60–69 | 718 (15.7) | 3856 (84.3) | 4574 (30.9) |  |
| 70–79 | 415 (8) | 4752 (92) | 5167 (34.9) |  |
| ≥80 | 81 (4) | 1924 (96) | 2005 (13.6) | <0.001 |
| **Year of diagnosis** |  |  |  |  |
| 2004 | 141 (18.6) | 618 (81.4) | 759 (5.1) |  |
| 2005 | 169 (19.5) | 696 (80.5) | 865 (5.8) |  |
| 2006 | 164 (16.8) | 814 (83.2) | 978 (6.6) |  |
| 2007 | 210 (17.4) | 1000 (82.6) | 1210 (8.2) |  |
| 2008 | 255 (14.9) | 1458 (85.1) | 1713 (11.6) |  |
| 2009 | 266 (13.5) | 1706 (86.5) | 1972 (13.3) |  |
| 2010 | 180 (12.1) | 1311 (87.9) | 1491 (10.1) |  |
| 2011 | 153 (12.6) | 1059 (87.4) | 1212 (8.2) |  |
| 2012 | 146 (12.2) | 1051 (87.8) | 1197 (8.1) |  |
| 2013 | 135 (11.8) | 1013 (88.2) | 1148 (7.8) |  |
| 2014 | 103 (9.1) | 1033 (90.9) | 1136 (7.7) |  |
| 2015 | 111 (9.9) | 1008 (90.1) | 1119 (7.6) | <0.001 |
| **Race** |  |  |  |  |
| White | 1885 (13.8) | 11804 (86.2) | 13689 (92.5) |  |
| Black | 73 (10.5) | 622 (89.5) | 695 (4.7) |  |
| Asian | 38 (23.2) | 126 (76.8) | 164 (1.1) |  |
| Others | 37 (14.7) | 215 (85.3) | 252 (1.7) | <0.001 |
| **Ethnicity** |  |  |  |  |
| Non-Hispanic | 1870 (13.7) | 11758 (86.3) | 13628 (97.3) |  |
| Hispanic | 64 (17.3) | 307 (82.7) | 371 (2.7) | 0.05 |
| **Insurance** |  |  |  |  |
| Private | 49 (12.6) | 339 (87.4) | 388 (2.6) |  |
| Non-Insured | 1070 (23.5) | 3492 (76.5) | 4562 (30.8) |  |
| Medicaid | 98 (15.6) | 529 (84.4) | 627 (4.2) |  |
| Medicare | 769 (8.6) | 8129 (91.4) | 8898 (60.1) |  |
| Other Government | 23 (13.9) | 142 (86.1) | 165 (1.1) |  |
| Insurance unknown | 24 (15) | 136 (85) | 160 (1.1) | <0.001 |
| **Charlson-Deyo Comorbidity Index** |  |  |  |  |
| 0 | 1594 (15.6) | 8641 (84.4) | 10235 (69.2) |  |
| 1 | 364 (10.6) | 3085 (89.4) | 3449 (23.3) |  |
| 2 | 60 (6.8) | 816 (93.2) | 876 (5.9) |  |
| 3 | 15 (6.3) | 225 (93.8) | 240 (1.6) | <0.001 |
| **Annual income, $** |  |  |  |  |
| <38000 | 243 (10.9) | 1985 (89.1) | 2228 (15.3) |  |
| 38000–47000 | 467 (12.2) | 3352 (87.8) | 3819 (26.3) |  |
| 48000–62000 | 534 (13) | 3566 (87) | 4100 (28.2) |  |
| ≥63000 | 744 (16.9) | 3656 (83.1) | 4400 (30.2) | <0.001 |
| **NAC** |  |  |  |  |
| No | 10907 (92.4) | 895 (7.6) | 11802 (78) |  |
| Yes | 3130 (94.2) | 194 (5.8) | 3324 (22) | 0.001 |
| **Facility type** |  |  |  |  |
| Community | 96 (10.6) | 813 (89.4) | 909 (6.2) |  |
| Comprehensive | 501 (10.2) | 4401 (89.8) | 4902 (33.3) |  |
| Academic/research | 1293 (17.3) | 6189 (82.7) | 7482 (50.8) |  |
| Integrated network | 110 (7.7) | 1321 (92.3) | 1431 (9.7) | <0.001 |
| **Education** |  |  |  |  |
| ≥21% | 249 (12.4) | 1763 (87.6) | 2012 (13.8) |  |
| 13–20.9% | 483 (12.8) | 3302 (87.2) | 3785 (26) |  |
| 7–12.9% | 676 (12.9) | 4560 (87.1) | 5236 (36) |  |
| <7% | 580 (16.5) | 2943 (92.3) | 3523 (24.2) | <0.001 |
| **Variant histology** |  |  |  |  |
| Pure urothelial | 1811 (13.8) | 11328 (86.2) | 13139 (88.8) |  |
| Adenocarcinoma | 19 (18.1) | 86 (81.9) | 105 (0.7) |  |
| Sarcomatoid | 32 (13.4) | 207 (86.6) | 239 (1.6) |  |
| Squamous | 47 (9) | 476 (91) | 523 (3.5) |  |
| Neuroendocrine tumour | 38 (16.7) | 190 (83.3) | 228 (1.5) |  |
| Urothelial with micropapillary component | 29 (16.4) | 148 (83.6) | 177 (1.2) |  |
| Others | 57 (14.7) | 332 (85.3) | 389 (2.6) | 0.02 |
| **Location (urban/rural)** |  |  |  |  |
| Rural | 130 (14.2) | 783 (85.8) | 913 (6.2) |  |
| Urban | 267 (10.6) | 2263 (89.4) | 2530 (17.1) |  |
| Metro areas | 1636 (14.4) | 9721 (85.6) | 11357 (76.7) | <0.001 |
| **T stage** |  |  |  |  |
| T2 | 1671 (14.5) | 9842 (85.5) | 11513 (77.8) |  |
| T3 | 221 (11.4) | 1712 (88.6) | 1933 (13.1) |  |
| T4 | 141 (10.4) | 1213 (89.6) | 1354 (9.1) | <0.001 |
| **N stage** |  |  |  |  |
| N0 | 1898 (13.9) | 11803 (86.1) | 13701 (92.8) |  |
| N1 | 62 (11.2) | 492 (88.8) | 554 (3.8) |  |
| N2–3 | 69 (13.4) | 447 (86.6) | 516 (3.5) | <0.001 |
